# Automatic thickness estimation for skeletal muscle in ultrasonography: evaluation of two enhancement methods

**DOI:** 10.1186/1475-925X-12-6

**Published:** 2013-01-22

**Authors:** Pan Han, Ye Chen, Lijuan Ao, Gaosheng Xie, Huihui Li, Lei Wang, Yongjin Zhou

**Affiliations:** 1 Shenzhen Institutes of Advanced Technology, Chinese Academy of Science, Shenzhen, China; 2 The Shenzhen Key Laboratory for Low-cost Healthcare, Shenzhen, China; 3 Second Affiliated Hospital of Kunming Medical University, Kunming, China; 4 School of Information Engineering, Nanchang Hangkong University, Nanchang, China

**Keywords:** Ultrasonography/Ultrasound imaging, Image enhancement, Gabor filtering, Multiscale vessel enhancement filtering, Muscle thickness

## Abstract

**Background:**

Ultrasonography is a convenient technique to investigate muscle properties and has been widely used to look into muscle functions since it is non-invasive and real-time. Muscle thickness, a quantification which can effectively reflect the muscle activities during muscle contraction, is an important measure for musculoskeletal studies using ultrasonography. The traditional manual operation to read muscle thickness is subjective and time-consuming, therefore a number of studies have focused on the automatic estimation of muscle fascicle orientation and muscle thickness, to which the speckle noises in ultrasound images could be the major obstacle. There have been two popular methods proposed to enhance the hyperechoic regions over the speckles in ultrasonography, namely Gabor Filtering and Multiscale Vessel Enhancement Filtering (MVEF).

**Methods:**

A study on gastrocnemius muscle is conducted to quantitatively evaluate whether and how these two methods could help the automatic estimation of the muscle thickness based on Revoting Hough Transform (RVHT). The muscle thickness results obtained from each of the two methods are compared with the results from manual measurement, respectively. Data from an aged subject with cerebral infarction is also studied.

**Results:**

It’s shown in the experiments that, Gabor Filtering and MVEF can both enable RVHT to generate comparable results of muscle thickness to those by manual drawing (mean ± SD, 1.45 ± 0.48 and 1.38 ± 0.56 mm respectively). However, the MVEF method requires much less computation than Gabor Filtering.

**Conclusions:**

Both methods, as preprocessing procedure can enable RVHT the automatic estimation of muscle thickness and MVEF is believed to be a better choice for real-time applications.

## Background

Ultrasonography, as an important medical image modality in the study of the musculoskeletal system, has been widely used to measure changes in muscle geometry, such as muscle thickness, muscle pennation angle, fascicle length and cross-sectional area [1-8], because it is versatile, inexpensive and radiation-free. More important, the change of muscle properties over time can form signals, representing architectural muscle behavior under contraction, namely sonomyography (SMG) [9-12]. Together with electromyography (EMG) [13-15], SMG can provide quantitative information of muscle contraction, help correlate the actions of several muscles, either agonistic or antagonistic, and subsequently advance medical and engineering efforts to understand, diagnose or even treat musculoskeletal disorders [5,16-20].

Specifically speaking, muscle thickness (MT), which can effectively reflect the muscle activities during muscle contraction [21-24], is an important measure for musculoskeletal studies using ultrasonography. There have been reports on relationships between MT and many other measures, such as level of physical activity [25], age [26], muscle stiffness [27] and response to exercise [28]. Meanwhile, for major muscles such as quadriceps and gastrocnemius muscle (GM), another important parameter, fascicle length (FL) can hardly be measured directly since it’s beyond the dimension of most ultrasound images. Therefore FL is usually based on MT and pennation angle (for example, [29,30]). However, MT measurement is traditionally based on manual selection of 2 reference points at the superficial and deep aponeuroses and usage of an on-screen caliper to obtain the MT frame by frame [31-34], which is time-consuming and subjective. There have been several studies targeting at automatic estimation of MT, such as the Revoting Hough Transform (RVHT) method [35,36], Radon Transform [37], Region of Interest (ROI) [24] and cross-correlation algorithm [38]. Taking RVHT as an example, with assumption that the superficial and deep aponeuroses could be located using RVHT as the very first 2 lines detected, the MT could be computed readily as the distance between them. However, besides the fact that the speckle noise associated with ultrasound image, sarcopenia or loss of muscle mass due to ageing, can affect the quality of the images obtained by ultrasound from deconditioned muscles [20]. This can make it difficult to accurately locate the borders of the muscles to obtain an accurate measure of the MT [39]. There have been two popular methods proposed to enhance the hyperechoic regions over the speckles in ultrasonography, namely Gabor Filtering [36,40,41] and Multiscale Vessel Enhancement Filtering (MVEF) [42,43]. In this paper, to investigate whether image enhancement can make sufficient preparation for automatic estimation of MT and which method is more appropriate for MT estimation, we will evaluate both Gabor Filtering and MVEF on gastrocnemius muscle images.

## Methods

**Figure 1:**
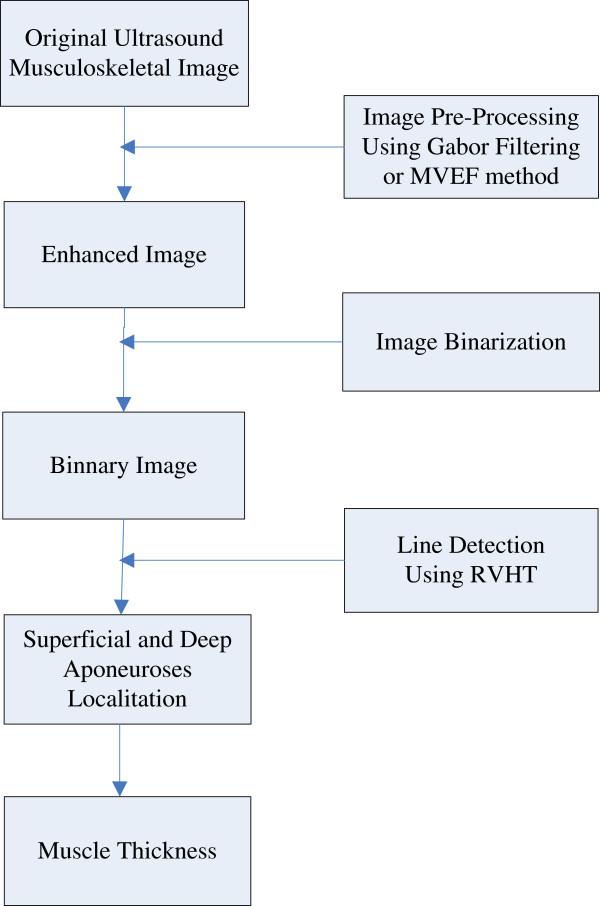
The automatic MT estimation procedure.

The automatic MT estimation procedure, based on RVHT method proposed previously, could include four steps: 1) image enhancement, 2) thresholding operation to generate black-white image and 3) locating of superficial and deep aponeuroses by RVHT and 4) computation of the distance between aponeuroses. The RVHT first computes accumulator matrix of Hough transform based on a black-white image called an “edge map”. This edge map represents the meaningful image contents. The RVHT method then locates the global maximum in the accumulator matrix of Hough transform that corresponds to the most dominant line-shaped feature points globally, using the standard Hough transform. Then the pixels close to the detected line are removed from the edge map and the Hough transform accumulator matrix is calculated again. The same procedure could be executed to search for another line [35]. For ultrasound images of skeletal muscles, usually the very first two lines detected would be the superficial and deep aponeuroses according to *a priori* knowledge. Finally the mean distance between each two lines detected by RVHT is computed, and the two lines which have maximum distance between them are recognized as the superficial and deep aponeuroses, and finally the mean distance between them is computed as the MT. Diagram for the procedures is shown in Figure 1.

### Gabor filtering

Taking into the account that the fascicles and fibroadipose spetas in a sonogram are tubular and include coherent orientation tendencies, the Gabor Filter bank method can be used to implement the image enhancement. The method includes three steps: the orientation filed estimation, frequency map computation and Gabor filtering confined by the orientation reliability. More details can be consulted in [36,44].

### Multiscale vessel enhancement filtering

The Multiscale Vessel Enhancement Filtering method is based on the second order local structure, with excellent noise and background suppression performance. The method also includes three steps: the Hessian matrix estimation (including the choice of Gaussian kernels), computation of eigenvector for each scale and processing for the maximum vesselness response. More details can be found in [42,43].

## Experiments

### Subjects

Three healthy male subjects (mean ± SD, age = 28.6 ± 0.6 years; body weight 67.0 ± 1.7 kg; height = 1.72 ± 0.01 m) volunteered to participate in this study. No participant had a history of neuromuscular disorders, and all were aware of experimental purposes and procedures. Human subject ethical approval was obtained from the relevant committee from the Hong Kong Polytechnic University, and informed consent was obtained from the subject prior to the experiment.

### Experiment protocol and data acquisition

The testing position of the subject was in accordance with the User’s Guide of a Norm dynamometer (Humac/Norm Testing and Rehabilitation System, Computer Sports Medicine, Inc., Massachusetts, USA). The subject was instructed to generate dorsiflexion/plantar-flexion movements in prone position. The torque was measured by the aforementioned dynamometer and muscle contracting in a range from 0 to 90% maximal voluntary contraction (MVC) is imaged by ultrasonography. 90% MVC is set as the highest value to avoid muscle fatigue.

**Figure 2:**
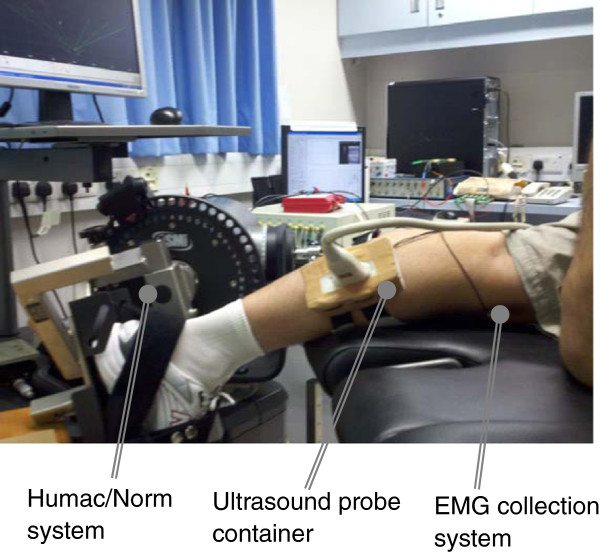
Experimental setup including EMG and ultrasound image data collection modules.

A real-time B-mode ultrasonic scanner (EUB-8500, Hitachi Medical Corporation, Tokyo, Japan) with an electronic linear array probe (EUP-L53L, 5.0-10.0 MHz Hitachi Medical Corporation, Tokyo, Japan) was used to obtain ultrasound images of muscles. The long axis of the ultrasound probe was arranged parallel to the long axis of the GM and on its muscle belly, decided accozrding to operator’s palpation. The ultrasound probe was fixed by a custom-designed foam container with fixing straps, and a very generous amount of ultrasound gel was applied to secure acoustic coupling between the probe and skin during muscle contractions, as shown in Figure 2. The probe was adjusted to optimize the contrast of muscle fascicles in ultrasound images. Then the B-mode ultrasound images were digitized by a video card (NI PCI-1411, National Instruments, Austin, USA) at a rate of 25 frame/s for later analysis.

### Data processing

The experiments detailed in last section ended up in 300 ultrasound images and all images were cropped to keep the image content only. All data were processed off-line using programs written in Matlab 7.11 (MathWorks, Inc., Massachusetts, USA) and the correlation and agreement analysis were performed using the computer package SPSS/PC 4.0 (SPSS Inc, Chicago, USA).

### Experiment setup for an aged subject with cerebral infarction

One male subject with unilateral limb dysfunction caused by cerebral infarction (age = 68 years; body weight = 71 kg; height = 1.72 m; right leg dysfunctional) volunteered to participate in this study. The human subject ethical approval was obtained from the relevant committee in Shenzhen Institutes of Advanced Technology, Chinese Academy of Sciences before carrying out the experiment. The subject was briefed about the procedure of experiment and written consents were collected prior to the experiment. During measurement, the subject performed plantarflextion both in left leg (Normal) and in right leg (Abnormal). A laptop ultrasound system (SS-10, Sonostar Technologies Co., Limited, Guangzhou, China), with a 7.5 MHz electronic linear array probe, was used to obtain the dynamic ultrasonic image sequences. The long axis of the probe was arranged parallel to the long axis of the gastrocnemius muscle. 64 consecutive frames from both legs are captured.

## Results

**Figure 3:**
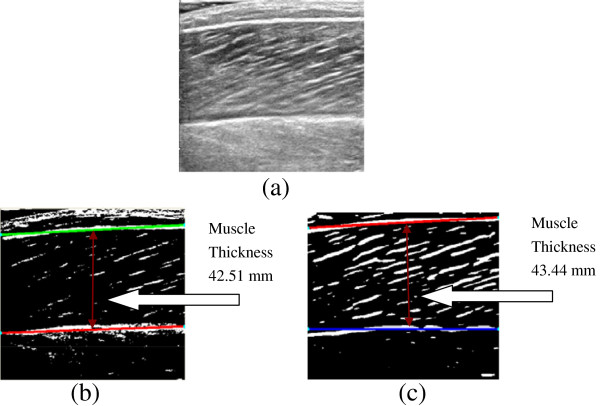
**The experimental results on a representative ultrasound image.** (**a**) the cropped image, (**b**) the line detection using RVHT after Gabor Filtering, (**c**) the line detection detection using RVHT after MVEF method. And the further MT estimation results are labeled in Figure 3b and (**c**).

In this section, experimental results from the comparison between the performance of the Gabor Filtering method and the one of the MVEF method over a series of clinical gastrocnemius muscle ultrasound images were first presented. The originally acquired images (640 × 480 pixels) were first cropped to keep the image content only, and the cropped images (403 × 373 pixels) were then enhanced by Gabor Filtering and MVEF methods respectively and binarized, as shown in Figure 3a-c. It should be noted that, in Gabor Filtering, empirically the default value of the reliability of the local orientation of image was set to 50%, which means that the corresponding location would be ignored unless when the reliability of the orientation is higher than 50%. The superficial and deep aponeuroses were then located by RVHT and MT was estimated automatically, as shown in Figure 3b-c. For all 300 frames, after either Gabor Filtering or MVEF method, the superficial and deep aponeuroses had been detected by RVHT as the very first two lines, as expected.

**Figure 4:**
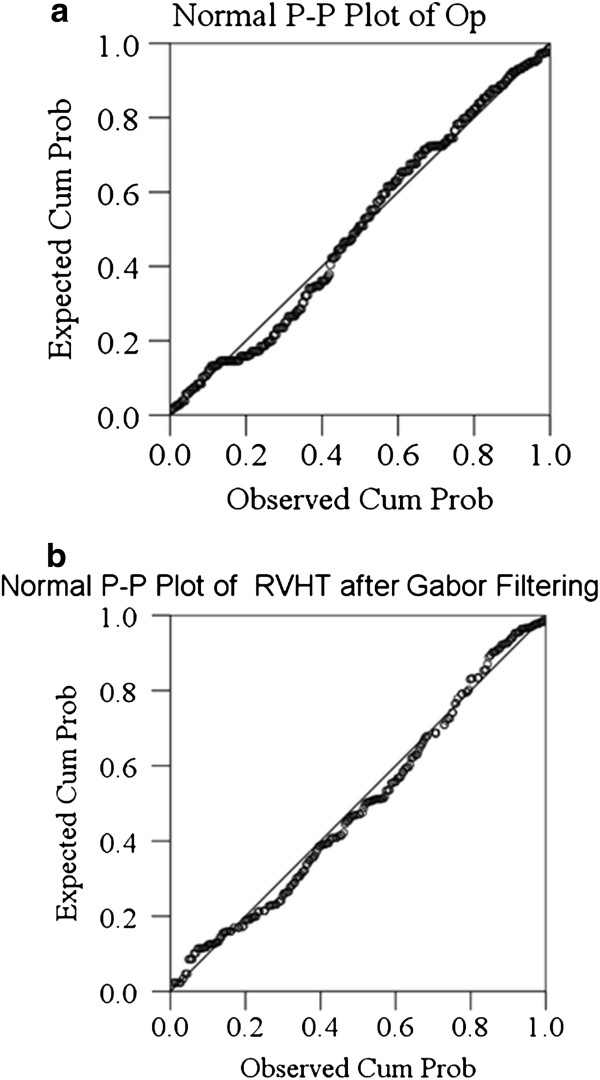
The normal P-P plots of three MT estimation results by (a) the manual operation results, (b) RVHT based on Gabor Filtering, (c) RVHT based on MVEF method.

**Figure 5:**
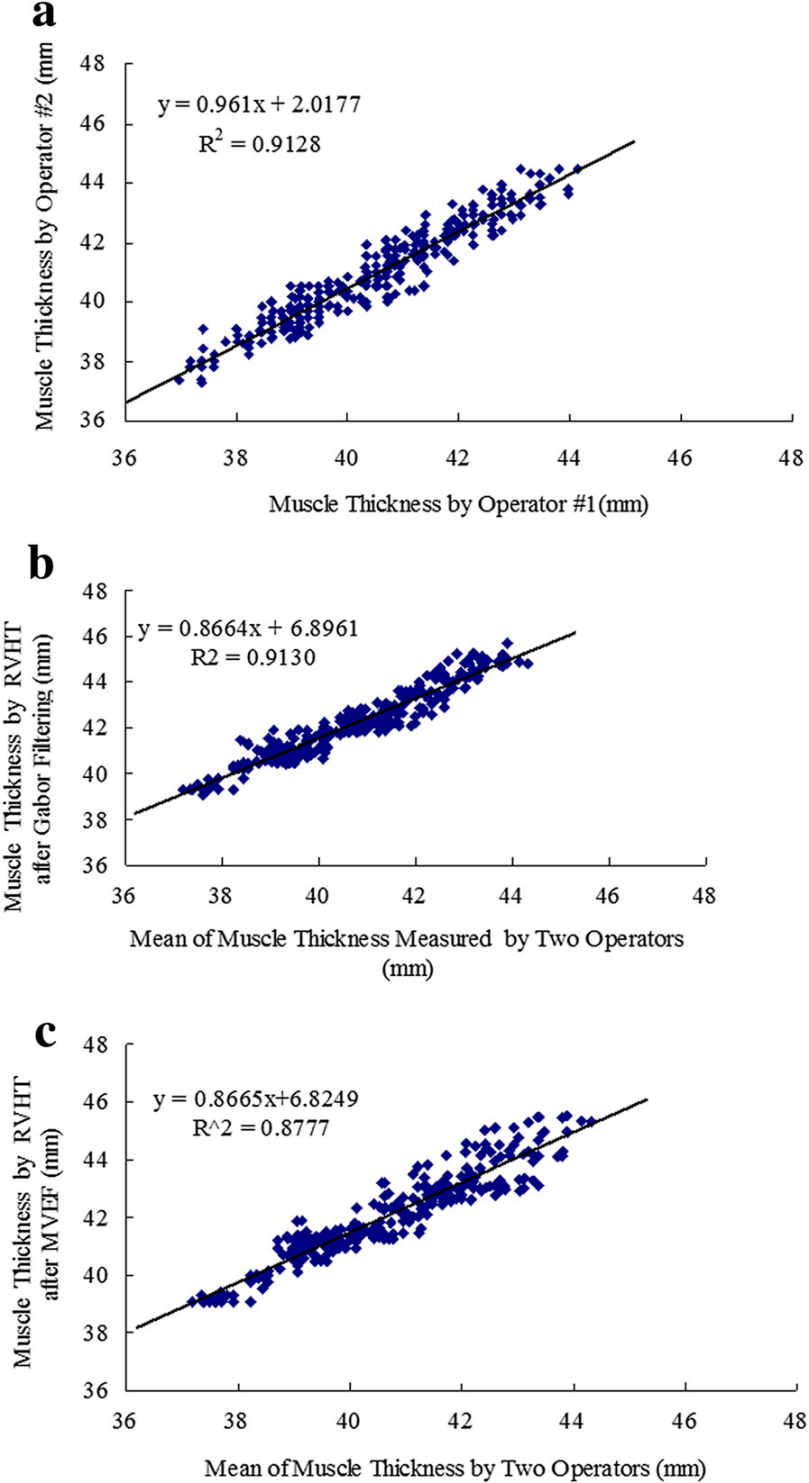
The comparison of MT estimation results, (a) between two operators, (b) using RVHT after Gabor Filtering and those by manual drawing, (c) using RVHT after MVEF method and those by manual drawing.

**Figure 6:**
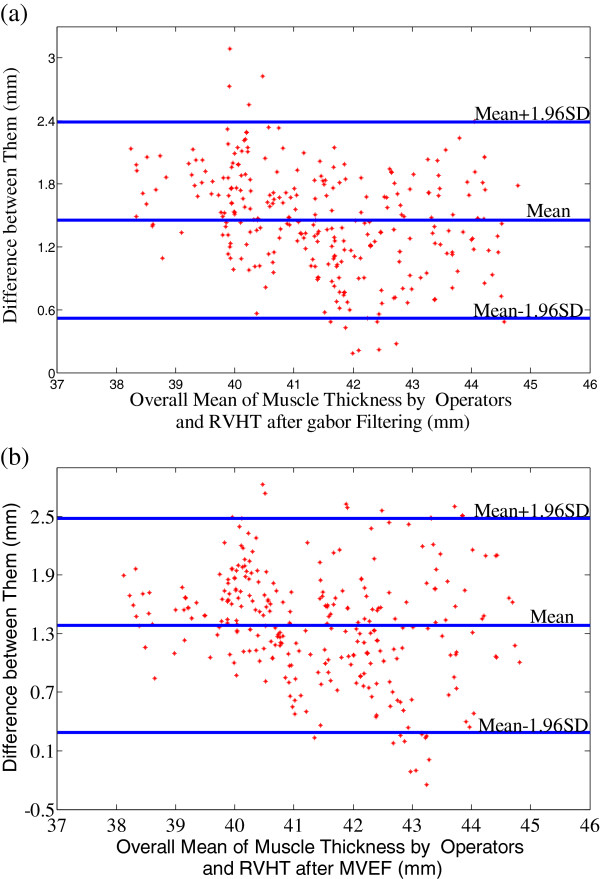
**The Bland-Altman plots.** (**a**) The Bland-Altman plot of the mean MT estimated manually by the two operators and the results obtained using RVHT after Gabor Filtering, (**b**) The Bland-Altman plot of the mean MT estimated manually by the two operators and the results obtained using RVHT after MVEF method.

In order to make a quantitative evaluation of the performance of the MT estimated by RVHT after the two methods, two operators were asked to manually draw lines and read MT of the 300 images digitally using a software (NIH Image, National Institutes of Health, USA). The superficial and deep aponeuroses were located independently and blindly in each image. And then the mean of the two operators’ results, named as “Op”, was considered to be a close estimation of the true value of MT. The P-P plots of the results obtained by manual drawing and the two automatic methods are shown in Figure 4a-c, suggesting that roughly estimated, the results data were all normally distributed. Therefore, based on the current recommendations for reliability analysis [45,46], inter-class correlation coefficient, intra-class correlation coefficient (ICC) and Bland-Altman plots were used to further explore the results. The correlation analysis of Op and the corresponding values estimated by RVHT after the two enhancement methods are all shown in Figure 5a-c. The square of the Pearson product moment inter-class correlation coefficients of the above three comparison study were 91.3%, 91.3% and 87.8% respectively. Figure 6 presents the details of the MT statistics using Bland-Altman plots [47]. The differences between the manually-obtained results and the corresponding values obtained by using RVHT after Gabor Filtering and MVEF method were 1.45 ± 0.48 and 1.38 ± 0.56 mm, respectively. The difference between the results obtained by RVHT after Gabor Filtering and MVEF method was 0.07 ± 0.54 mm. The computation of ICCs [48] was conducted using the SPSS, based on a single rating, absolute-agreement, 2-way mixed effect model. The standard error of the measurements (SEMs) was also calculated to quantify the measurement precision (i.e., $\mathrm{SEM}=s_{x}\sqrt{1-\mathrm{ICC}}$ where s_*x*_ is the standard deviation). The minimal detectable changes (MDCs) were calculated as $\mathrm{SEM}\times 1.96\times \sqrt{2}$, which represents the minimal change in MT that must occur to be 95% confident that a true change occurred [38]. The mean values ± SDs, ICC, SEMs, and MDCs in each subject are presented in Table 1. The measurements based on RVHT after Gabor Filtering decreased the SEM by 12.9% as that of MVEF method. The MDCs based on RVHT after Gabor Filtering and MVEF method were 2.3% vs. 2.2%, 1.3% vs. 2.3%, 1.5% vs. 1.3% of the MT, respectively. The use of the mean among 3 subjects as an outcome measure resulted in MDCs of 1.8% (Gabor Filtering) and 2.1% (MVEF method) of the MT, respectively.

**Table 1:** MeanSD, estimation reliability, SEM, and MDC of the thickness measurements

**Subject**	**Mean ± SD (mm)**	**ICC**	**95% confidence interval**	**SEM (mm)**	**MDC (mm)**
Results based on RVHT after Gabor Filtering					
S1	42.02 ± 1.63	0.953	0.929-0.968	0.35	0.97
S2	41.61 ± 1.56	0.985	0.978-0.990	0.19	0.53
S3	40.77 ± 1.62	0.982	0.974-0.988	0.22	0.61
Overall	41.47 ± 1.69	0.975	0.968-0.980	0.27	0.75
Results based on RVHT after MVEF					
S1	41.93 ± 1.57	0.955	0.932-0.969	0.33	0.91
S2	41.71 ± 1.64	0.956	0.935-0.971	0.34	0.94
S3	40.66 ± 1.57	0.984	0.977-0.990	0.20	0.55
Overall	41.43 ± 1.68	0.966	0.957-0.973	0.31	0.86

**Figure 7:**
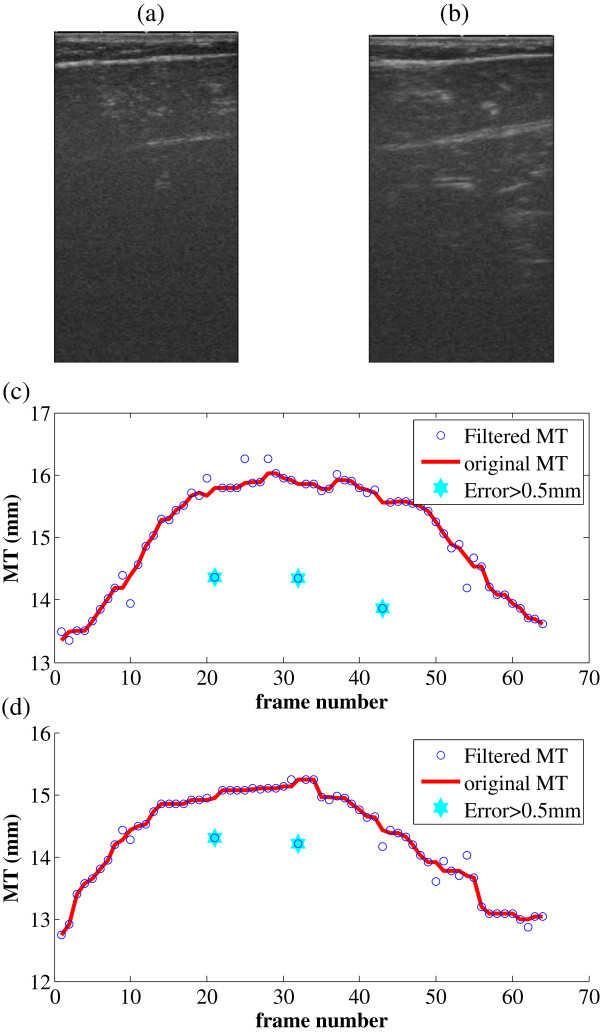
**Results on a patient with unilateral limb dysfunction caused by cerebral infarction.** (**a**) a typical frame from the 64 frames for left leg, (**b**) a typical frame from the 64 frames for right leg(dysfunctional), (**c**) the MTs estimated, using the proposed method with MVEF, for the left leg performing a pantarfelxion exercise, (**d**). the MTs estimated, using the proposed method with MVEF, for the right leg performing a pantarfelxion exercise.

Two typical frames from left and right legs of the aged subject are shown in Figure 7a-b. MTs estimated using the proposed method with MVEF are shown in Figure 7c-d. For both group of data, in 5 out of total 128 (64 for each leg) frames, the MTs estimated are significantly different from the neighboring values. To have a better observation on the transitions of MT for both legs during the plantarflextion excercises, simple median filters were used to render the smooth versions. Frame number, corresponding to MTs with difference > 0.5 mm between the original and smooth versions are, are also marked out in Figure 7. MT for the left and right legs are 15.06 ± 0.86 and 14.30 ± 0.76 mm respectively.

## Discussion

As both Gabor Filtering and MVEF are established methods for skeletal muscle enhancement [36,43], in this work we focus on their performance as a pre-processing step of automatic estimation of MT. It’s hoped that this study would provide consultative guide for widespread application of the computerized MT estimation, thus to replace the time-consuming and subjective manual drawing.

First of all, to estimate the MT automatically, we use RVHT to detect lines in ultrasound images of skeletal muscle, and the superficial and deep aponeuroses are expected to be the very first two lines detected. Without enhancement procedure before RVHT, the performance of aponeuroses detection is quite poor, indicated by the fact that the superficial and deep aponeuroses could not be located as the very first 2 lines in 110 of 300 images. However, as mentioned before, it becomes 300 out of 300 images after either image enhancement method. In other words, for the automatic estimation of MT, we must drop the assumption that the superficial and deep aponeuroses are the strongest lines in ultrasound images, unless a proper image enhancement procedure is used.

As for the quantitative performance of the two methods, MT estimated by RVHT after the two methods have high correlation (R^2^ = 91.3% and 87.8% respectively) with small differences (1.45 ± 0.48 and 1.38 ± 0.56 mm respectively, when MT is about 38-46 mm) to manual drawing measurement. And Bland-Altman plots in Figure 6 also show the good agreement between the MT estimated by RVHT after the two methods and manual results.

It should be noted that, as shown in Figure 6, the differences of MT estimated by RVHT after either Gabor Filtering or MVEF method and that obtained by manual drawing is about 1.4 mm, and only in 3 out of 300 images the manual results were larger than RVHT method after MVEF, and none is larger after Gabor Filtering. It’s believed that this bias is due to the fact that in manual method, MT was calculated as the border/nearest distance between the superficial and deep aponeuroses in the middle of the ultrasound image at a 90-degree angle from the deep aponeurosis [33], while in automatic method using RVHT, the MT will be estimated as roughly the middle-line distances between superficial and deep aponeuroses. In other words, by nature there is this bias due to the different acting definitions of MT between the manual and automatic method using RVHT. We have calculated the thickness of superficial aponeuroses using active contour for the 300 images and the result is about 1.51 ± 0.05 mm, which could further reduce the difference/bias and add a piece of proof to the deduction that the bias is mainly caused by the mean thickness of aponeuroses themselves. On the other hand, we’d like to point out that although the mean results of two operator is taken to be the ‘true’ thickness, the two operators also, not surprisingly as shown in Figure 5a, do not output identical results of MT and the difference is 0.44 ± 0.48 mm.

The time costs of the two methods to process one image were about 5.1 s and 0.3 s in Matlab, respectively, suggesting that MVEF is more suitable for real-time applications where MT transitions are often interested.

The results on the aged subject with cerebral infarction further demonstrated the feasibility of the proposed method as an automatic method for muscle MT estimation. Interestingly the dysfunctional right leg exhibited smaller MT compared to left leg, by 94.9% and 89.1%, in terms of mean and standard deviation respectively. We’d like to note that the current report is the first one to prove the feasibility of automatic measurement of MT in longitudinal direction, with the help of image enhancement techniques. The automatic measurement of MT then can make possible the continuous monitoring of the time course of MT along with muscle contraction. It’s objective and not labor-intensive, especially when the images to analyze are numerous.

In the further research, more musculoskeletal images should be collected including those under pathological conditions. Problems are expected to arise, for example, acute patients usually can not adopt positions such as standing, or prone lying to allow optimal access to measurement sites, which may increase the likelihood of measurement errors [39]. And the corresponding further improvements on the automatic estimation of MT would certainly broaden the application area of ultrasound imaging in clinical musculoskeletal system, such as, study of the relationship between MT changes and tumbling for Tumble Monitoring and Identification (TMI) [49].

## Conclusions

In conclusion, this study aims at automatic estimation of skeletal MT using RVHT, and an evaluation of two image enhancement methods in ultrasound images, as a preprocessing step to make sure that superficial and deep aponeuroses can be detected by RVHT as the very first 2 line-shaped features. Experimental results from 300 images in total showed that, both methods can provide effective preparation for RVHT and to further estimate MT. Specifically speaking, the superficial and deep aponeuroses could not be located by RVHT as the very first 2 lines in 110 of 300 images without image enhancement. However, as mentioned before, it becomes 300 out of 300 images after either image enhancement method. Meanwhile, the results from both methods have high correlation and great agreement with manually-obtained results, while the MVEF runs much faster. This study demonstrated that with the help of proper image enhancement preprocessing procedures, RVHT can be used for automatic estimation of skeletal MT.

## Abbreviations

SMG: Sonomyography; EMG: Electromyography; MT: Muscle thickness; GM: Gastrocnemius muscle; FL: Fascicle length; RVHT: Revoting hough transform; MVEF: Multiscale vessel enhancement filtering; ICC: Intra-class correlation coefficient.

## Competing interests

The authors declare that they have no competing interests.

## Authors’ contributions

PH: analysed the data and composed the manuscript together with YZ, YZ: proposed the idea. LO, YC, HL, GX and LW performed experiments, processed the data and made the discussions. All authors read and approved the final manuscript.
